# Complete genome sequence of *Rhodococcus qingshengii* strain R isolated from Antarctic soil

**DOI:** 10.1128/mra.01316-24

**Published:** 2025-05-06

**Authors:** Xin Wang, Hongyan Wang, Huijuan Jin, Hengyi Liao, Xinyue Qian, Manman Zhang, Zhen Weng, Erik Hoffnagle, Xuhao Wang, Jiaojiao Yang, Jingjing Wang, Yiru Cui, Xiuying Li, Xiaodong Liu, Xin Chen, Yi Yang

**Affiliations:** 1 Key Laboratory of Pollution Ecology and Environmental Engineering, Institute of Applied Ecology, Chinese Academy of Sciences 74763 , Shenyang, Liaoning, China; 2 University of the Chinese Academy of Sciences 74519, Beijing, China; 3 Water Resources Research Center, University of Hawaii at Manoa 58575, Honolulu, Hawaii, USA; 4 Department of Civil, Environmental and Construction Engineering, University of Hawaii at Manoa, Honolulu, Hawaii, USA; 5 Bloomage Biotechnology Corporation, Tianjin, China; 6 School of Pharmaceutical Engineering, Shenyang Pharmaceutical University 58575, Shenyang, Liaoning, China; 7 Anhui Province Key Laboratory of Polar Environment and Global Change, School of Earth and Space Sciences, University of Science and Technology of China 12652, Hefei, Anhui, China; 8 Key Laboratory for Polar Ecosystem and Climate Change of Ministry of Education, School of Oceanography, Shanghai Jiao Tong University 12474, Shanghai, China; 9 Key Laboratory of Forest Ecology and Silviculture, Institute of Applied Ecology, Chinese Academy of Sciences 74763 , Shenyang, Liaoning, China; University of Southern California, Los Angeles, California, USA

**Keywords:** *Rhodococcus*, facultative anaerobes, fermentation, Antarctica, iron reduction

## Abstract

We report the complete genome sequence of *Rhodococcus qingshengii* strain R, isolated from Antarctic soil. The genome consists of a 6,361,549-bp chromosome and two plasmids (99,385 bp and 312,136 bp). The genome encodes 5,915 protein-coding sequences, 56 tRNAs, and five copies each of the 5S, 16S, and 23S rRNA genes.

## ANNOUNCEMENT

*Rhodococcus qingshengii* strain R was isolated from a microcosm established with a sample of 5 g soil collected in 2010 from Beaufort Island in Antarctica (166° 58' 23.6" E, 76° 58' 23.6" S, depth 20.5 cm). Isolation was performed through serial dilution plating on bicarbonate-buffered (30 mM) basal mineral salt medium supplemented with 0.5 g/L peptone, 5 mM lactate, and 10 mM ferric citrate at 4°C under anoxic conditions that mirrored those used in the microcosm setup (1). Although *Rhodococcus* is a typical soil inhabitant (2), it has been found in diverse habitats (3) and oligotrophic environments (4). Isolated from the extreme environment of Antarctica, strain R exhibits Fe(III) reduction potential, suggesting unique metabolic capabilities that may influence iron cycling in polar ecosystems. This combination of geographic origin and functional significance makes strain R an ideal model for advancing our understanding of microbial processes in extreme environments.

Genomic DNA was extracted using the cetyltrimethylammonium bromide (CTAB) method from cells grown statically under the conditions described above (5, 6), and the same DNA preparation was used for both PacBio and Illumina library constructions. Sequencing was performed using Illumina NovaSeq 6000 and PacBio II sequencer (Pacific Biosciences, Menlo Park, CA). For PacBio sequencing, DNA was sheared using g-TUBEs (Covaris, USA), generating fragments of approximately 8–10 kb for long-insert library preparation. The fragments were ligated by hairpin adapters via SMRTbell Express Template Prep Kit v 2.0 (Pacific Biosciences, Menlo Park, CA) (7). After that, Sequel II System Chemistry 2 was used in sequencing, and SMRT Link v10.1 was used to control raw read quality, correct error, and trim adapter. For Illumina sequencing, a DNA library with an average insert size of 350 bp was constructed using the NEBNext Ultra DNA Library Prep Kit from New England Biolabs (USA). The library was then sequenced on an Illumina NovaSeq 6000 instrument (Illumina Inc., USA) with a paired-end approach (2 × 150 bp) (8). Quality control was performed using Filtlong v0.2.1 (9) and Fastp v0.23.4 (10) for PacBio and Illumina reads, respectively. The genome was assembled on the Galaxy platform using Unicycler v0.47 (11) (12), integrating 114,003 filtered PacBio reads and 5,794,688 filtered Illumina reads. The assembly indicated that both the chromosome and the smaller plasmid were circular, as determined by overlapping sequence ends. Genome completeness and contamination were subsequently assessed using CheckM v1.1.3 (13). All software was run with default parameters. Functional annotation was conducted with the NCBI Prokaryotic Genome Annotation Pipeline (PGAP) (14).

The final assembly includes a 6,361,549 bp circular chromosome and two plasmids: one circular plasmid of 99,385 bp and one linear plasmid of 312,136 bp (Table 1). Unicycler classified the two additional contigs as plasmids based on their size, structural features, and the detection of plasmid-associated genes. The chromosome contains 6,073 predicted protein-coding genes, 56 tRNAs, and five copies each of the 5S rRNA, 16S rRNA, and 23S rRNA genes. The circular and linear plasmids encode 103 and 376 sequences respectively, with no RNA genes. The genome was identified as *R. qingshengii* based on an average nucleotide identity (ANI) of 98.79% to the reference genome of *Rhodococcus qingshengii* JCM 15477 (accession no. PRJDB938) (15).

**TABLE 1:** Genome features of strain R

Feature	Chromosome	Circular plasmid	Linear plasmid
Assembly length (bp)	6,361,549	99,385	312,136
G + C content (%)	62.5	62.4	61.8
No. of assembled contigs	2	1	1
No. of coding sequences	5,946	103	376
No. of tRNAs	56	0	0
No. of rRNAs (5S, 16S, 23S)	5,5,5	0,0,0	0,0,0
GenBank accession no.	CP151090	CP151091	CP173713
BioSample accession no.	SAMN40861310	SAMN40861310	SAMN40861310
BioProject accession no.	PRJNA1097065	PRJNA1097065	PRJNA1097065
Genome completeness	99.94%	-^*a*^	-
Contamination	1.17%	-	-
Coverage	203×	167×	396×

^*a*^ -, not applicable.

## Data Availability

The genome sequence has been deposited in GenBank under accession numbers CP151090 (chromosome), CP151091 (circular plasmid), and CP173713 (linear plasmid). Raw sequences are available in SRA (SRR31020090 for Illumina, SRR31020089 for PacBio). The BioSample and BioProject accession numbers are SAMN40861310 and PRJNA1097065, respectively
